# Internalization and Down-Regulation of the ALK Receptor in Neuroblastoma Cell Lines upon Monoclonal Antibodies Treatment

**DOI:** 10.1371/journal.pone.0033581

**Published:** 2012-03-30

**Authors:** Pierre Mazot, Alex Cazes, Florent Dingli, Joffrey Degoutin, Théano Irinopoulou, Marie-Claude Boutterin, Bérangère Lombard, Damarys Loew, Bengt Hallberg, Ruth Helen Palmer, Olivier Delattre, Isabelle Janoueix-Lerosey, Marc Vigny

**Affiliations:** 1 Université Pierre et Marie Curie, Paris, France; 2 Institut National de la Santé et de la Recherche Médicale, UMRS, Paris, France; 3 Institut du Fer à Moulin, Paris, France; 4 Institut Curie, Centre de Recherche, Paris, France; 5 Institut National de la Santé et de la Recherche Médicale, Institut Curie, Paris, France; 6 Institut Curie, Centre de Recherche, Laboratoire de Spectrométrie de Masse Protéomique, Paris, France; 7 Department of Molecular Biology, Umea University, Umea, Sweden; The Institute of Cancer Research, United Kingdom

## Abstract

Recently, activating mutations of the full length ALK receptor, with two hot spots at positions F1174 and R1275, have been characterized in sporadic cases of neuroblastoma. Here, we report similar basal patterns of ALK phosphorylation between the neuroblastoma IMR-32 cell line, which expresses only the wild-type receptor (ALK^WT^), and the SH-SY5Y cell line, which exhibits a heterozygous ALK F1174L mutation and expresses both ALK^WT^ and ALK^F1174L^ receptors. We demonstrate that this lack of detectable increased phosphorylation in SH-SY5Y cells is a result of intracellular retention and proteasomal degradation of the mutated receptor. As a consequence, in SH-SY5Y cells, plasma membrane appears strongly enriched for ALK^WT^ whereas both ALK^WT^ and ALK^F1174L^ were present in intracellular compartments. We further explored ALK receptor trafficking by investigating the effect of agonist and antagonist mAb (monoclonal antibodies) on ALK internalization and down-regulation, either in SH-SY5Y cells or in cells expressing only ALK^WT^. We observe that treatment with agonist mAbs resulted in ALK internalization and lysosomal targeting for receptor degradation. In contrast, antagonist mAb induced ALK internalization and recycling to the plasma membrane. Importantly, we correlate this differential trafficking of ALK in response to mAb with the recruitment of the ubiquitin ligase Cbl and ALK ubiquitylation only after agonist stimulation. This study provides novel insights into the mechanisms regulating ALK trafficking and degradation, showing that various ALK receptor pools are regulated by proteasome or lysosome pathways according to their intracellular localization.

## Introduction

Full-length anaplastic lymphoma kinase (ALK) is a tyrosine kinase receptor (RTK) originally identified in human and mouse [1], [2]. Orthologues of this receptor have also been identified in *Drosophila* and *Caenorabditis elegans* [3], [4]. In vertebrates, the ALK receptor is expressed transiently during development of central and peripheral nervous systems strongly suggesting implication in development and function in this system. However ALK functions in vertebrates remain poorly understood and in those organisms the nature of the cognate ligand is still highly controversial [see [5] for a review].

Deregulation of ALK signaling has been associated with development of various cancers [see [6] for a review]. The intracellular portion (containing the kinase domain) of ALK was firstly identified as part of the nucleophosmin (NPM)-ALK oncogenic fusion protein resulting from chromosomal translocation frequently associated with anaplastic large cell lymphoma (ALCL). Other chromosomal translocations implicating intracellular portion of ALK fused with different partners have been associated with various cancers, such as EML4-ALK involved in 5% of non-small-cell lung cancer (NSCLC) [see [6] for a review]. The ALK receptor has emerged as an attractive target for the development of anticancer therapeutic strategy [see [7] for a review] and recently crizotinib (also known as PF-2341066) has given hopeful results in clinical trials for the treatment of NSCLC [8].

The full length receptor ALK has been shown to be implicated in somatic and germinal cases of neuroblastoma through gene amplification and/or activating point mutations [9], [10], [11], [12], [13]. Two hot spots of mutations at positions F1174 and R1275 have been identified in sporadic cases. Using stably transfected NIH3T3 cells expressing mutated ALK at position F1174 or R1275, we recently established that the constitutive kinase of the mutated receptors impaired their trafficking, with retention in intracellular compartment [14]. Strikingly intracellular mislocalized receptors were significantly less phosphorylated when compared with the cell surface pool, and normal receptor trafficking could be restored by abolition of its activity using the specific ALK kinase inhibitor NVP-TAE 684 (TAE) [14]. While small molecule inhibitors are one of the most promising strategies for the treatment of cancers linked to RTKs, the development of monoclonal antibodies (mAb) may also be highly valuable [15]. Moreover, combinations of tyrosine kinase inhibitors and antibodies are already used in therapeutic treatments [16]. We previously generated a range of mAbs against the extracellular part of the ALK receptor and isolated mAbs with either agonist or antagonist mAb characteristics [17]. The full characterization of the effects of such antibodies on the properties of the ALK receptor, including stability and trafficking is an important prerequisite to their potential use in clinical practice.

In this study we demonstrate that the ALK^F1174L^ receptor endogenously expressed in SH-SY5Y cells is largely retained in an intracellular compartment in a poorly phosphorylated state where it is rapidly degraded by the proteasome. We subsequently show that addition of agonist antibody induces ALK activation, internalization, ubiquitylation, and lysosomal degradation of cell surface receptor. In contrast, antagonist antibody, which dimerizes ALK without activation, leads to internalization and plasma membrane recycling.

## Results

### ALK receptor phosphorylation, localization and degradation in neuroblastoma SH-SY5Y cells

#### ALK phosphorylation in SH-SY5Y and IMR-32 neuroblastoma cell lines

**Figure 1:**
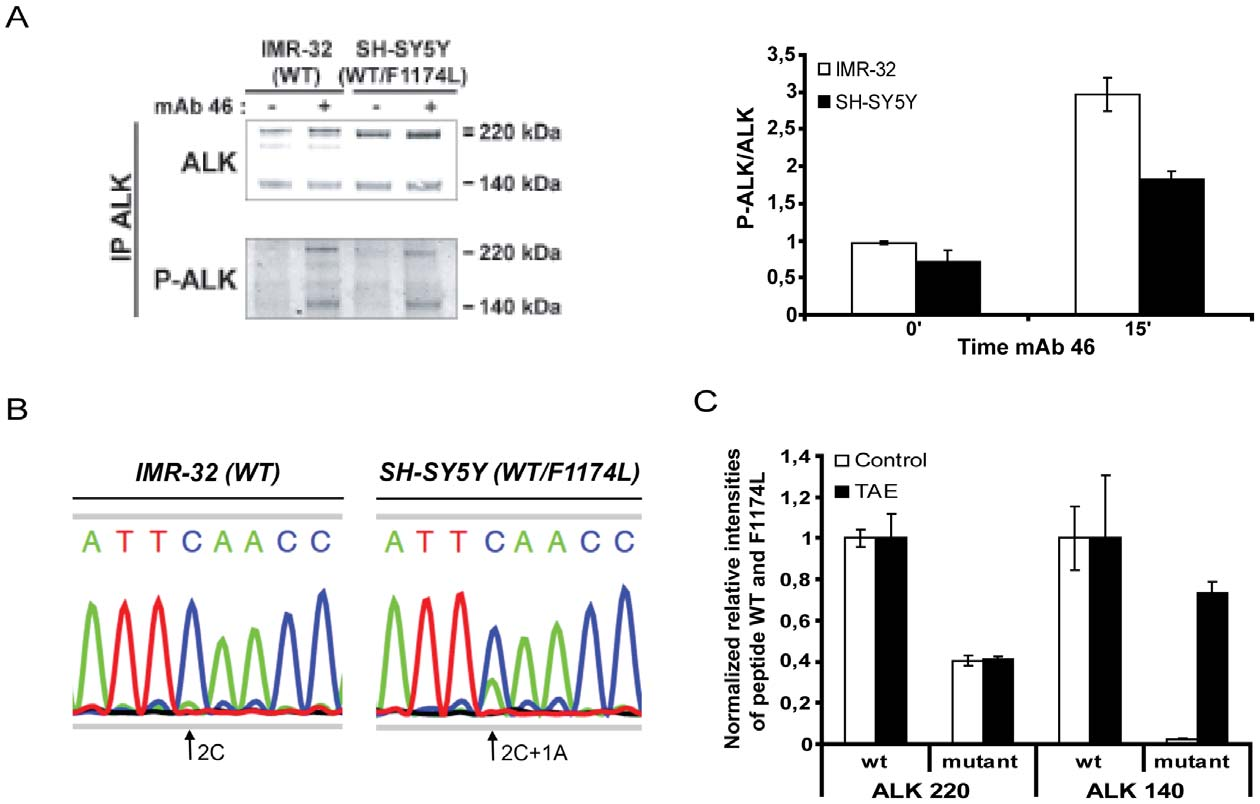
ALK^WT^ and ALK^F1174L^ expression and phosphorylation in the SH-SY5Y neuroblastoma cell line. **A.** IMR-32 (WT) and SH-SY5Y (WT/F1174L) cells were untreated or stimulated with 6 nM agonist mAb 46 for 15 min. ALK immunoprecipitates were immunoblotted with polyclonal anti-ALK (REAB) and antiphosphotyrosine (4G10 platinium). *Right panel*: Quantification of the ratio P-ALK/ALK in IMR-32 and SH-SY5Y cells, results are expressed in mean and s.e.m. **B.** Total RNA of IMR-32 (WT) and SH-SY5Y (WT/F1174L) were extracted and cDNA were obtained by reverse transcription. Sequencing chromatograms of the cDNAs obtained after RT-PCR are shown. **C.** SH-SY5Y cells (WT/F1174L) were treated or not with the ALK specific tyrosine kinase inhibitor NVP-TAE684 at 50 nM for two days. After ALK immunoprecipitation proteomics analysis and quantifications of interested peptides (carrying or not the mutation spot) were done by ESI-MS, after normalization.

We first investigated the impact of ALK mutation on receptor phosphorylation by comparing two neuroblastoma cell lines, IMR-32 cells expressing only wild-type receptor and SH-SY5Y cells exhibiting a heterozygous ALK F1174L activating mutation [11], [12]. As expected, in both cell lines, a doublet at 220 kD corresponding to the full length receptor, together with a band representing the cleaved form at 140 kD [17] were detected. The level of ALK expression in the two cell lines was roughly similar and ALK phosphorylation was barely discernible in either cell lines under basal conditions, even given, the presence of an activated mutation in the SH-SY5Y cell line (Fig. 1A). Agonist mAb stimulation performed with the mAb 46 induced an increase of ALK phosphorylation in both IMR-32 and SH-SY5Y cells (Fig. 1A, right panel: representative Western Blot, left panel: quantification). In agreement with our previous data [18] we observed an induction of ALK phosphorylation for the upper band of the 220 kD doublet and the 140 kD form.

#### Analysis of the RNA and protein levels of ALK WT and F1174L mutant in the SH-SY5Y neuroblastoma cell line

Since no increase in the basal phosphorylation of the ALK receptor could be detected in SH-SY5Y cells, we sought to measure the relative ratio of the WT and mutant ALK receptors in this sample. Sequencing of cDNA generated from total RNAs confirmed that IMR-32 cells express only ALK^WT^ (Fig. 1B); whereas in SH-SY5Y, we observed the presence of both ALK^WT^ and ALK^F1174L^ with a ratio of 2∶1 (Fig. 1B). In the parental SK-N-SH cell line from which the SH-SY5Y cell line has been derived, a trisomy of chromosome 2p including the *ALK* locus has been observed, with two wild-type alleles for one mutated one (I. Janoueix-Lerosey, unpublished observations). It is likely that the SH-SY5Y cell line bears a similar 2p gain that would be consistent with the proportion of ALK^WT^ and ALK^F1174L^ mRNAs observed here. We next investigated the ratio of ALK^WT^ and ALK^F1174L^ receptors for the 220 kD and 140 kD forms by mass spectrometry in SH-SY5Y cells. After tryptic digestion and normalization using synthetic peptides we could detect the peptide containing or not the mutation site for both the 220 kD and the 140 kD forms (Figures S1A and S1B). We first analyzed the 220 kD forms and observed a ratio of more than two ALK^WT^ for one mutated receptor. In contrast, the 140 kD form contained only ALK^WT^ (Fig. 1C).

#### Kinase inhibition restored cell surface localization of the mutated receptors in SH-SY5Y cells

We previously demonstrated intracellular retention of activated ALK in NIH3T3 cells stably transfected with ALK^F1174L^ and showed that kinase inhibition restored maturation and cell surface localization of the mutated receptors [14]. The lack of ALK^F1174L^ in the 140 kD form in SH-SY5Y could therefore be explained by the same intracellular trafficking defect in this cell line, i.e. retention of ALK^F1174L^ in the ER/Golgi compartments. We therefore treated SH-SY5Y cells with TAE, a small-molecule ALK inhibitor and then performed a *label-free* quantitative proteomics study of WT and F1174L mutated ALK as described above, both for the 220 kD and 140 kD forms. TAE treatment led to a strong increase of the amount of ALK^F1174L^ present in the 140 kD form, demonstrating the rescue of the normal intracellular trafficking of the mutated receptor (Fig. 1C).

#### Proteasomal degradation of the intracellular pools of ALK^WT^ and ALK^F1174L^

**Figure 2:**
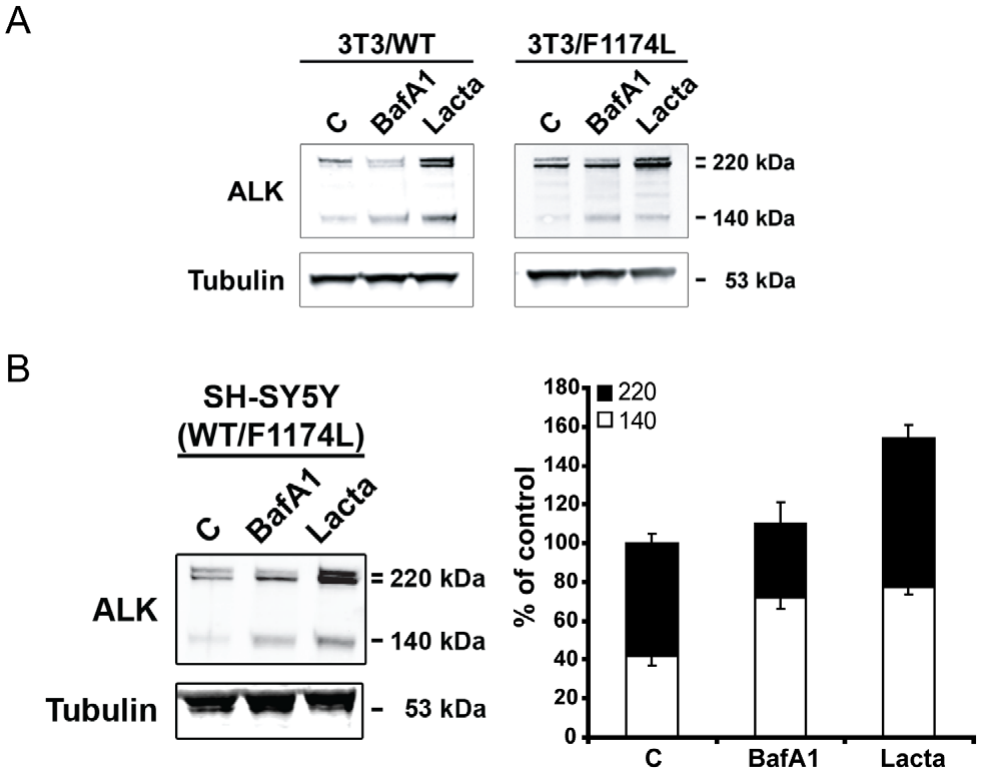
Proteasome dependent degradation of receptor retained in intracellular compartment. **A.** NIH3T3 cells stably expressing either the WT ALK or F1174L mutated ALK were non-treated or treated with Bafilomycin A1 (0.25 µM) or with Lactacystin (10 µM) for 16 hours. ALK immunoprecipitates from 1 mg of total cell lysate proteins were subjected to western blot analysis. ALK was immunoblotted with polyclonal REAB antibody. **B.** SH-SY5Y were non-treated or treated with Bafilomycin A1 (0.25 µM) or with Lactacystin (10 µM) for 16 hours. ALK immunoprecipitates from 1.5 mg of total cell lysate proteins were subjected to western blot analysis using polyclonal REAB antibody. The experiment was done in triplicates and quantified with LiCor Odyssey system. Results were expressed in percentage of control +/− s.e.m.

In order to gain insight into the degradation mechanisms involved in the regulation of ALK stability, we explored the two main protein degradation pathways, i.e. the proteasome and lysosome pathways. We took advantage of NIH3T3 cells stably expressing only either ALK^WT^ (3T3/WT) or ALK^F1174L^ (3T3/F1174L) and used lactacystin or bafilomycin A1 to specifically inhibit proteasome or lysosome dependent degradation, respectively. In 3T3/WT cells, bafilomycin A1 treatment led to the enrichment of the 140 kD form of ALK correlating with the decrease of the upper band of the 220 kD doublet (Fig. 2A). These two products have been shown previously to be located at the plasma membrane. The effect of bafilomycin A1 treatment on 3T3/F1174L cells was hardy detectable. In contrast, in both cell lines, lactacystin treatment led to an increase of the lower band of the 220 kD doublet that was previously shown to be an intracellular form of the receptor and an increase in the total amount of ALK was also observed (Fig. 2A). These results therefore indicate that the intracellular pools of ALK, either ALK^WT^ or ALK^F1174L^, are preferentially degraded by the proteasome whereas the turn-over of the ALK receptor located at the plasma membrane is achieved by lysosomes.

In SH-SY5Y cells, biotinylation experiments confirmed that the upper band of the 220 kD doublet as well as the 140 kD form were located at the cell surface, whereas the lower band of the 220 kD doublet was intracellular (Figure S2). We investigated the effect of lactacystin or bafilomycin A1 drugs on SH-SY5Y cells. Bafilomycin A1 induced a decrease of the upper band of the 220 kD doublet correlated with an increase of the 140 kD form and lactacysin led to a strong increase of ALK level for both the 220 kD and the 140 kD forms (Fig. 2B, left panel: representative blot, right panel: quantification). These observations are consistent with the results obtained in the 3T3 cells and indicate that, in this neuroblastoma cell line, the intracellular pools of ALK, including both ALK^WT^ and ALK^F1174L^ receptors are degraded by the proteasome.

### ALK trafficking upon agonist or antagonist mAb treatment

#### Agonist and antagonist mAb treatments induced ALK internalization

**Figure 3:**
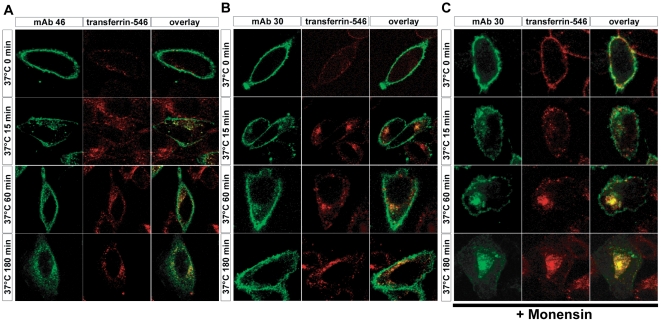
Agonist mAb treatment induced lysosome targeting and antagonist mAb treatment induced cell surface recyling of ALK receptor. **A.** CHO cells were transiently transfected with construct encoding for ALK WT. Cells were pulsed 10 minutes on ice at 4°C with 6 nM of agonist mAb 46 and transferrin coupled to AlexaFluor 546, then chased at 37°C in a time course manner. Direct immunodetection of agonist mAb 46 were done with anti-mouse secondary antibody coupled to Alexa Fluor 488. Cellular localization of ALK receptors were shown by immunofluorescence using confocal laser scanning microscopy. Merge images were mounted thanks to Photoshop software. **B.** CHO cells were transiently transfected with construct encoding for ALK wild type. Cells were pulsed 10 minutes on ice at 4°C with 6 nM of antagonist mAb 30 and transferring coupled to AlexaFluor 546, then chased at 37°C in a time course manner. Direct immunodetection of antagonist mAb 30 were done with anti-mouse secondary antibody coupled to Alexa Fluor 488. Cellular localization of ALK receptors were shown by immunofluorescence using confocal laser scanning microscopy. Merged images were mounted thanks to photophop software. **C.** Experiment identical to B but with a cell pre-treatment with monensin (50 µM) for 30 min.

We first determined whether agonist mAb could trigger ALK internalization. The low level of ALK expression in neuroblastoma cell lines did not allow us to follow internalization by immunofluorescence. We thus employed transiently transfected ALK^WT^ in CHO cells. To investigate the internalization of ALK primarily located at the membrane before treatment, we incubated living cells with agonist mAb 46 for 10 min at 4°C in order to block endocytosis. We then chased cells at 37°C and observed the resultant, in order to allow receptor trafficking. Using confocal microscopy we were able to detect mAb 46 bound to the ALK receptor. As expected, after incubation at 4°C we could only detect the mAb∶ALK receptor complex at the cell surface. After 15′ of incubation at 37°C ALK appeared intracellularly, and after 3 h the staining was essentially intracellular (Fig. 3A).

Having demonstrated that agonist mAb was able to trigger ALK internalization, we next investigated the effects of antagonist mAb treatment. The antagonist antibodies dimerize ALK without activation [17]. After 15′ of incubation at 37°C with mAb 30, ALK appeared intracellularly (Fig. 3B). Upon longer exposure (3 h), the receptor was detected internally but a significant fraction of ALK was still located at the plasma membrane in contrast with the effect observed when employing the agonist mAb (Fig. 3B compared to Fig. 3A).

#### Differential intracellular trafficking of ALK in response to agonist or antagonist mAb

To characterize differences in ALK trafficking in response to agonist and antagonist mAb we further investigated the receptor intracellular trafficking after internalization. Indeed it is well established that after ligand induced internalization, RTK could be either addressed to lysosome for degradation, or recycled to plasma membrane through trafficking in specific endosomal compartments.

We first performed a co-localization study of internalized ALK after agonist mAb 46 treatments with a marker of recycling endosomes, transferrin. For this purpose CHO cells transiently transfected with ALK^WT^ were incubated for 10 min at 4°C with both agonist mAb 46 and transferrin-546, then cells were replaced at 37°C for indicated times. After 15 min, 1 h and 3 h, ALK partially co-localized with tranferrin-546 (Fig. 3A). Thus agonist mAb treatment induced only a partial receptor recycling to plasma membrane after internalization.

Antagonist mAb 30 treatment led to the internalization of ALK but an important fraction of ALK was still located at the plasma membrane. These results could be explained by plasma membrane recycling after internalization. Indeed internalized ALK in response to antagonist mAb strongly co-localized with transferrin (Fig. 3B). To fully confirm that antagonist mAb treatment induced ALK recycling after internalization, we used monensin a classical inhibitor of this process. Addition of 100 nM of monensin before mAb 30 treatment led to a strong accumulation of internalized ALK starting after 1 h of cell incubation at 37°C and ALK strongly co-localized with transferrin-546 (compared Fig. 3B and 3C). Thus, in response to antagonist mAb treatment, ALK was recycled to plasma membrane after internalization.

#### Agonist mAb treatment induced ALK degradation whereas antagonist mAb induced ALK internalization without down-regulation

**Figure 4:**
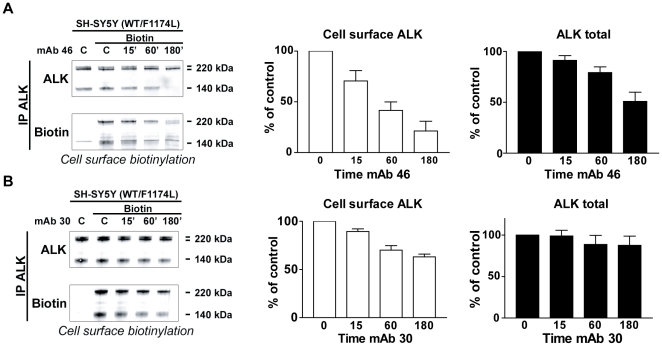
Agonist mAb treatment induced ALK degradation whereas antagonist mAb induced ALK internalization without down-regulation. **A.** SH-SY5Y cells were untreated (-) or treated with agonist mAb 46 for 15 min, 60 min or 3 hours. At the end of the agonist treatment, cells were subjected to cell surface protein biotinylation. ALK immunoprecipates from 1.5 mg of total cells lysate proteins were analyzed by western blot. Biotinalyted cell surface ALK were detected with streptavidin coupled to IRdye 800, and total ALK with REAB antibody. Experiments were done in triplicates, total and biotinylated (cell surface) ALK were quantified with LiCor Odyssey software. Results are expressed in percentage of control +/− s.e.m. **B.** SH-SY5Y cells were untreated or treated with agonist mAb 30 for 15 min, 1 h or 3 h. At the end of mAb treatment, cells were subjected to cell surface protein biotinylation. ALK immunoprecipates from 1.5 mg of total cells lysate proteins were analyzed by western blot. Biotinalyted cell surface ALK were detected with streptavidin coupled to IRdye 800, and total ALK with REAB antibody. Experiments were done in triplicates, total and biotinylated (cell surface) ALK were quantified with LiCor Odyssey software. Results are expressed in percentage of control +/− s.e.m.

Since both agonist and antagonist mAb treatment induced ALK internalization in CHO, we next investigated whether these treatments resulted in down-regulation of the receptor in neuroblastoma cell lines. We performed cell surface protein biotinylation experiments after exposure of the SH-SY5Y neuroblastoma cells to agonist or antagonist mAbs. Treatment with agonist mAb46 clearly induced a progressive decrease of biotinylated ALK with 80% of receptor internalized after 3 h (Fig. 4A, left panel: representative western Blot, middle panel: quantification of cell surface ALK). Interestingly the level of ALK decreased during mAb treatment. This effect was noticeable after 1 h and strikingly after 3 h (Fig. 4A, right panel: quantification of total ALK). On closer inspection the remaining receptor largely corresponded to the intracellular ALK i.e the pool of receptor not accessible to the mAb. Thus, ALK is internalized and then degraded in response to agonist mAb treatment. We next investigated the effect of antagonist mAb treatment on ALK internalization and degradation in SH-SY5Y cells. Similarly to experiments performed with the agonist mAb we performed cell surface protein biotinylation experiments exposure to mAb 30 (Fig. 4B, left panel: representative Western Blot, middle panel: quantification of cell surface ALK, right panel: quantification of total ALK). Treatment with mAb 30 also induced a progressive decrease of ALK biotinylation but reached a plateau with 40% of internalization between 1 h and 3 h (Fig. 4B) correlated with a weak decrease of total ALK. These results suggest that ALK dimerization without activation leads to receptor internalization followed by recycling to the plasma membrane.

#### Tyrosine kinase activity is required for ALK down-regulation in neuroblastoma cell lines

**Figure 5:**
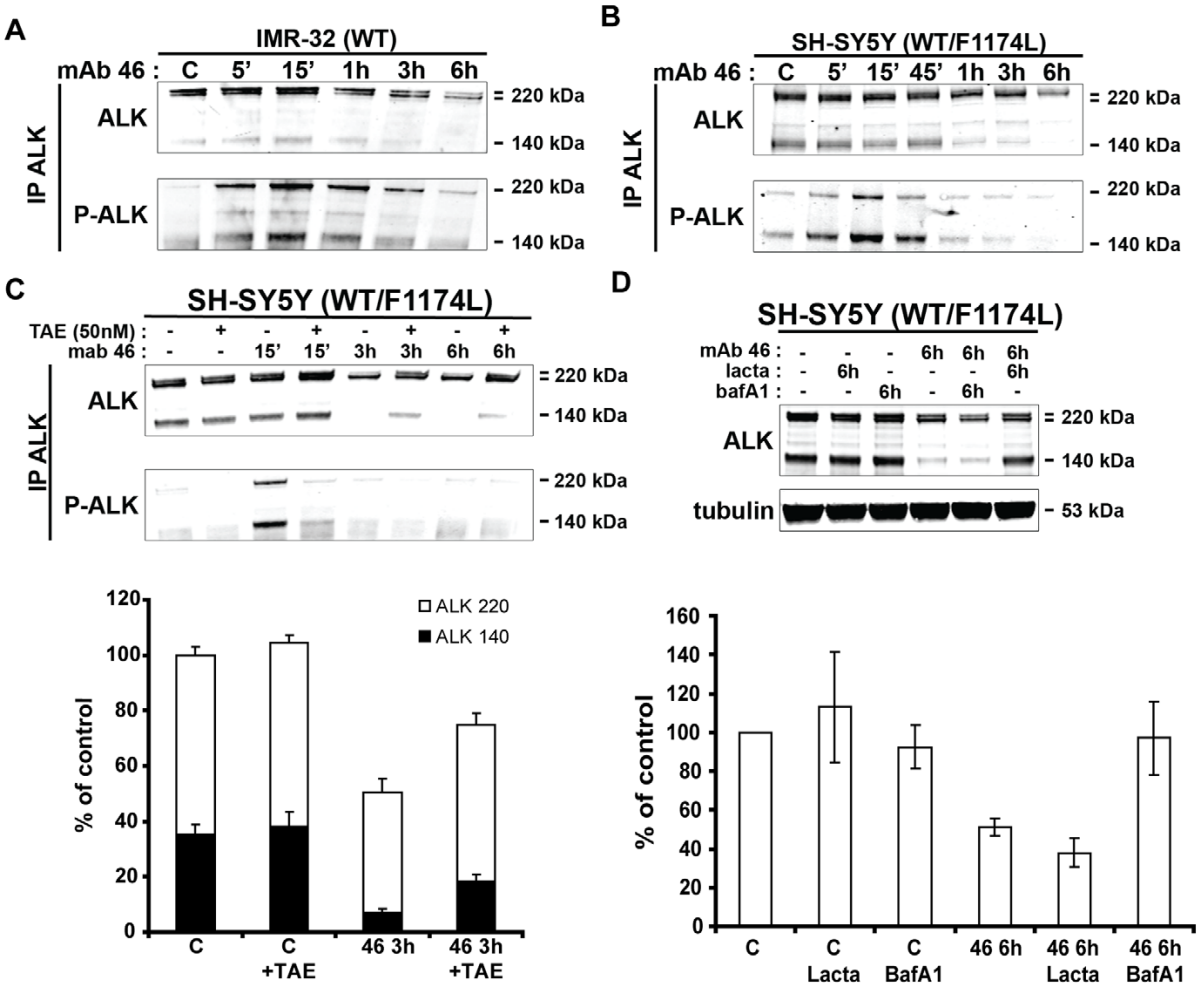
ALK down-regulation after agonist mAb 46 treatment. **A. IMR-32 (WT) and B. SH-SY5Y (WT/F1174L)** were treated with mAb 46 for 15 min to 6 h. ALK immunoprecipitates from 1.5 mg of total cell lysate proteins were submitted to western blot. Total ALK were immunoblotted with polyclonal REAB and phosphorylated ALK were detected with monoclonal phosphotyrosine antibody 4G10 platinium. Tubulin acted as a loading control. **C.** SH-SY5Y cells were pre-treated or not with 50 nM NVP-TAE684 one hour before cell stimulation by agonist mAb 46 at 6 nM in time course manner (0 min to 6 h). ALK immunoprecipitates from 1.5 mg of total cell lysate proteins were submitted to western-blot analysis and immunoblotted as described. *Lower panel*: Experiments were done in triplicates and the 220 kD forms (the doublet was impossible to separate) and the 140 kD form of ALK were quantified with LiCor Odyssey software. Results are expressed in percentage of control +/− s.e.m. **D.** SH-SY5Y cells were pre-treated with either bafilomycin (0.25 µM) or lactacystin (10 µM) for 15′, then non treated or treated with agonist mAb 46 at 6 nM during 6 hours. ALK immunoprecipitates from 1.5 mg of total cell lysate proteins were submitted to Western-blot analysis as described. *Lower panel:* Experiments were done in triplicates total ALK (220 kD forms+140 kD form) was quantified with LiCor Odyssey software. Results are expressed in percentage of control +/− s.e.m.

We further investigated the ALK down-regulation process induced by agonist mAb. For this purpose, we treated IMR-32 and SH-SY5Y with mAb 46 over a 6 h time course. We then followed receptor activation and degradation by western blot after immunoprecipitation. In both cell lines, agonist mAb 46 induced ALK phosphorylation with a peak at 15 min. ALK down-regulation led to a decrease of the receptor phosphorylation with slower kinetics for IMR-32 cells as compared toSH-SY5Y cells (Fig. 5A and Fig. 5B). In both cases receptor degradation started after 1 h of agonist mAb treatment and increased with time. ,As expected the lower 220 kD form of ALK, which corresponds to the intracellular pool of receptor, was much less sensitive to agonist mAb induced down-regulation.

We then investigated the importance of kinase activity in the degradation of ALK. As expected, pretreatment with the inhibitor TAE led to a strong inhibition of ALK phosphorylation induced by mAb46 treatment (Fig. 5C, upper panel: representative Western Blot, lower panel: quantification). Interestingly, TAE treatment strongly inhibited the agonist induced ALK degradation of both the 220 kD and 140 kD forms (Fig. 5C). Thus, ALK activation is essential to trigger ALK down-regulation.

We then tested which protein degradation pathway was involved in ALK degradation after agonist mAb stimulation. SH-SY5Y cells were pre-treated with either lactacystin or bafilomycin A1 for 15 min and then treated with agonist mAb 46 for 6 h. We selected this duration of treatment since it triggers an almost complete down regulation of cell surface ALK both in IMR-32 and SH-SY5Y cells (Fig. 5A). This treatment was much shorter than the one used in the experiment described in Fig. 2A. Proteasome inhibition by lactacystin had no effect on agonist induced ALK degradation. On contrary bafilomycin A1 treatment completely abolished ALK down-regulation after agonist treatment (Fig. 5D, upper panel: representative Western Blot, lower panel: quantification). These results demonstrated that after agonist mAb treatment, ALK was mainly addressed to lysosome for degradation, with a minor pool of receptors recycled to plasma membrane.

#### ALK activation is required for Cbl recruitment and receptor ubiquitylation

Ubiquitylation of RTK has been demonstrated to be a critical event in the regulation of intracellular sorting and lysosome targeting [19] and the ALK receptor has been previously showed to recruit the ubiquitin ligase Cbl after activation [20]. We therefore investigated whether differential ALK intracellular trafficking in response to mAb is related to the recruitment of Cbl and receptor ubiquitylation.

**Figure 6:**
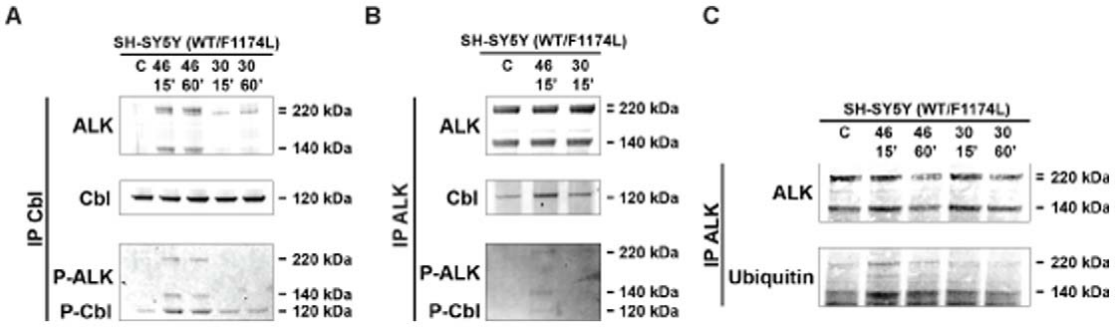
Kinase activation dependent down-regulation was regulated by Cbl ubiquitin ligase and ALK ubiquitylation. **A.** SH-SY5Y cells were treated or not with either agonist mAb 46 or antagonist mAb 30 at 6 nM during 15 or 60 min. Cbl immunoprecipitates from 2 mg total cell lysate proteins were submitted to western-blot analysis, and then immunoblotted with polyclonal anti Cbl and ALK recruitment with polyclonal REAB antibody. Phosphorylated ALK and Cbl were revealed with 4G10 antibody. **B.** SH-SY5Y cells were serum starved for 16 hours and non-treated or treated with either agonist mAb 46 or antagonist mAb 30 at 6 nM during 15 or 60 min. ALK immunoprecipitates from 2 mg total cell lysate proteins were submitted to western-blot analysis and then immunoblotted with polyclonal REAB. Cbl recruitment was revealed with polyclonal anti Cbl antibody. Phosphorylated ALK and Cbl were revealed with 4G10 antibody. **C.** SH-SY5Y were serum starved for 16 hours, and then non-treated or treated with either mAb 46 or mAb 30 at 6 nM for 15 minutes or 60 minutes. ALK immunoprecipitates were submitted to Western-blot analysis as described.

We first performed Cbl immunoprecipitation, examining co-immunoprecipitation of ALK by immunoblotting in SH-SY5Y cells. In control conditions we could not detect ALK co-immunoprecipitation with Cbl. Agonist mAb treatment led to co-immunoprecipitation of Cbl and ALK (Fig. 6A). Furthermore, we also detected the phosphorylation of both proteins strongly suggesting that the phosphorylation of Cbl resulted from ALK activation. We then tested Cbl recruitment after ALK immunoprecipitation. After ALK immunoprecipitation we detected a weak recruitment of Cbl in control conditions; however agonist mAb treatment strongly increased this interaction (Fig. 6B). Once again, agonist mAb activation triggered ALK and Cbl phosphorylation. Finally we tested ALK ubiquitylation in response to mAb treatment in SH-SY5Y cells. For this purpose we performed ALK immunoprecipitation after mAb treatment and investigated ALK ubiquitylation using anti ubiquitin antibody P4D1. We detected a basal ALK ubiquitylation, which was moderately but significantly increased by agonist mAb 46 stimulation. Altogether these results demonstrated that ALK activation by agonist mAb stimulation induced receptor phosphorylation thus allowing Cbl recruitment and receptor ubiquitylation (Fig. 6C), which is required for lysosome targeting.

## Discussion

### In neuroblastoma cell lines SH-SY5Y ALK^F1174L^ is intracellular and hypophosphorylated

One of the striking results of this study was the lack of discernible difference in the ALK phosphorylation pattern in basal conditions as well as after mAb agonist stimulation in IMR-32 cells (ALK^WT^) and SH-SY5Y cells (ALK^WT^/ALK^F1174L^). This result, however, did not implicate that the mutated receptor does not exhibit any basal constitutive activity. *In Vitro* kinase assay in SH-SY5Y and IMR-32 cells confirmed higher kinase activity of the ALK^F1174L^ compared to the ALK^WT^ (Data not shown). We also demonstrated that treatment with TAE684 inhibitor is able to rescue normal trafficking of ALK^F1174L^ in SH-SY5Y cells, as previously established in other models [12]. *In Vitro* kinase assay and rescue of normal trafficking of the mutated receptor by kinase inhibitor TAE, strongly suggest that lack of basal ALK phosphorylation in SH-SY5Y cells is not reliable to a defect of kinase activity of the mutated receptor.

Ours results on ALK phosphorylation pattern in neuroblastoma cell line required two major comments. First, we used the general phosphorylated tyrosine antibody 4G10 to detected ALK phosphorylation. We could not exclude that ALK F1174L mutation induced receptor phosphorylation on specific tyrosines that are not recognized by the 4G10 antibody. However, detection of ALK increased phosphorylation after agonist mAb treatment confirmed that 4G10 is a valuable antibody to detect ALK phosphorylation. An investigation in depth of ALK activation and autophosphorylation mechanism induced by activating mutation in neuroblastoma cell line appears very important to better understand its role in neuroblastoma proliferation.

Second, our results illustrated that ALK^F1174L^ expression are not necessarily link to increased ALK phosphorylation in neuroblastomas. SH-SY5Y neuroblastoma cell lines could not be representative to all the neuroblastoma cells bearing ALK^F1174L^ mutation, and further studies of ALK phosphorylation with larger neuroblastoma samples and neuroblastoma cell lines will be required to confirm our results. However, comparison of ALK phosphorylation in SH-SY5Y and IMR-32 cells shed light on requirement to carefully investigate ALK phosphorylation in neuroblastoma, even after activating mutation identification.

It has been suggested that ALK activation and transforming capacity may be variable according to the mutation type. In particular, the ALK^F1174L^ receptor has been shown to exhibit a higher oncogenic potential compared to the ALK^R1275Q^ receptor [21]. Interestingly, we previously demonstrated that the ALK^R1275Q^ receptor was proportionally more localized at the plasma membrane than the ALK^F1174L^ mutant [14]. Both the mutation type and the expression level may therefore impact basal phosphorylation of ALK receptor and oncogenic potential of the mutated receptors.

Interestingly, in SH-SY5Y cells, we detected at least twice as much ALK^WT^ than ALK^F1174L^ full length ALK receptor at 220 kD. Furthermore our analysis showed that the form at 140 kD did not contain any ALK^F1174L^ indicating that the full length form of the mutated receptor essentially did not reach the membrane. Thus the truncated form at 140 kD appears to be a valuable marker of normal receptor trafficking. The mutated receptor was essentially retained in intracellular compartments with very low level of basal phosphorylation in agreement with previous data obtained in other cell systems [14]. Importantly, ALK inhibition by TAE684 restored normal trafficking of mutated receptor in this cell line as demonstrated by the detection by mass spectroscopy of ALK^F1174L^ in the 140 kD form after TAE treatment. The absence of detectable phosphorylation of the mutated ALK receptor may rely on a permanent dephosphorylation by specific tyrosine phosphatases. Finally, we document that intracellular pools of ALK are degraded by the proteasome. Since kinase activation of ALK^F1174L^ likely leads to its intracellular retention this phenomenon is particularly relevant for the mutated receptor.

### ALK internalization and intracellular trafficking in response to agonist and antagonist antibodies

In this report, we demonstrate for the first time that the full length ALK receptor can be internalized in response to both agonist and antagonist mAb. Indeed, it has been shown previously that a chimeric EGFR-ALK protein composed by the extracellular and transmembrane parts of the EGFR and the intracellular part of ALK [21] was neither internalized nor down-regulated in response to EGF, on contrary to the EGFR [22]. We demonstrate that the ALK receptor, upon activation by agonist mAb, is internalized followed by down-regulation in a lysosome dependent degradation. In contrast, antagonist mAb treatment induces ALK receptor internalization through addressing to recycling compartments. The EGFR-ALK chimera therefore does not fully mimic the native ALK receptor. ALK has been previously demonstrated to be able to recruit the ubiquitin ligase Cbl after mAb agonist stimulation [20]. We demonstrate that differences in ALK intracellular trafficking induced by agonist or antagonist mAb is due to the recruitment of Cbl by activated ALK, which in turn leads to ALK ubiquitylation. In the case of the EGF receptor endocytosis, the respective requirement of receptor dimerization, ubiquitylation and/or kinase activation is still controversial [23], [24], [25], [26]. Our results on ALK internalization in response to antagonist mAb support the hypothesis that dimerization rather than kinase activity or ubiquitylation, is sufficient to induce receptor internalization.

### ALK degradation mechanism is dependent of receptor subcellular localization

In this work, we establish two different mechanisms of ALK degradation, depending on its cellular localization. The down-regulation of the cell surface ALK receptor resulting from its activation by agonist mAb is achieved by the lysosome. The recruitment of the ubiquitin ligase Cbl and ALK ubiquitylation appears to be essential regulatory events for triggering degradation by the lysosome. In contrast, the degradation of the ALK receptor retained in intracellular compartments relies on the proteasome. This is true in particular for the ALK^F1174L^ mutant retained in the ER/Golgi compartments as a consequence of its constitutive activity. Intracellular retention induced by constitutive activation has already been described for others RTK and particularly investigated in case of the Flt3-ITD receptor [27]. A minor pool of cell surface Flt3-ITD receptors are degraded through Cbl dependent ubiquitylation [28], whereas intracellular Flt3-ITD receptors are degraded by the proteasome by a largely unknown mechanism. Our data point out the importance of deciphering the proteasome targeting mechanism of misslocalized and constitutively activated RTK. Hsp90 inhibitors and HDAC inhibitors treatment, by stimulation of proteasome dependent degradation of misslocalized RTK in intracellular compartment, lead to cell proliferation inhibition [29]. Proteasome dependent degradation of mutated ALK suggests that use of Hsp90 inhibitors or HDAC inhibitors could be an attractive strategy to inhibit ALK dependent neuroblastoma proliferation.

The ALK receptor is an attractive therapeutic target in several human cancers, including neuroblastoma in which the full length ALK molecule may be activated by point mutations. This study provides novel insights into the mechanisms regulating sub-cellular localization and metabolism of full length receptor and deciphers more precisely the properties of mAbs on ALK activity and trafficking. Interestingly, anti-ALK mAb defined as agonist antibodies since they rapidly and transiently induced receptor phosphorylation were shown to trigger ALK degradation when cells were treated for several hours. We documented that ALK activity and localization are regulated by complex mechanisms. Further experiments will allow to determine the effects of antibodies targeting the ALK receptor on cellular proliferation and survival and establish whether they may be useful for a therapeutic issue in patients affected with neuroblastoma.

## Materials and Methods

### Cell lines

NIH 3T3 and SH-SY5Y cells were purchased from ATCC. IMR-32 and CHO cells were a kind gift of Dr. P. Kogner and Dr. C. Faure respectively. The transfected 3T3/WT and 3T3/F1174L cell lines were previously described [14]. CHO, IMR-32 and SH-SY5Y cells were maintained respectively with F12 medium, minimum essential medium (MEM) and F12/MEM (v/v) supplemented with 1× non-essential amino acids and 1 mM sodium pyruvate. Cell media were supplemented with 10% foetal calf serum (Invitrogen, Cergy-Pontoise, France).

### Antibodies and reagents

We used the following commercial antibodies were: Mouse antiphosphotyrosine antibody 4G10 platinium (Millipore) mouse anti-ubiquitine antibody P4D1 and rabbit anti-Cbl (Santa Cruz , Heidelberg, Germany)

Rabbit polyclonal anti-ALK (REAB) and monoclonal antibodies anti-ALK (mAb 15, 30, 46, 48) have been previously described [17]. The selective ALK kinase inhibitor NPV-TAE684 was synthesized following the formula described in [30]. Lactacystin, Bafilomycin A1, Monensin were purchased from Sigma-Aldrich, St Quentin Fallavier, France.

### Western blotting and immunoprecipitation

Cells were serum starved for 16 hours and then treated with 1 µg/ml of mAb 46, or mAb 48 and with 4 µg/ml of mAb 30. Cells were treated with 50 nM of NVP-TAE684 for 48 h or 30 min before the addition of mAbs. Lactacystin and Bafilomycin were used respectively at 10 µM and 0.25 µM for 16 hours in basal conditions or 15 min in pretreatment before mAb treatment.

Cells were washed rapidly with cold phosphate buffer saline (PBS) buffer (containing 5 mM sodium fluoride and 100 mM sodium orthovanadate) and were lysed in a cold RIPA buffer as described in [18]. Lysate were clarified by centrifugation at 21 000 *g* for 10 min at 4°C. Protein concentration was determined by the method of Bradford using the micro BCA Protein Assay Reagent Kit (Pierce). Immunoprecipitation and Western-blot analysis were performed as described in [14].

### Biotinylation assay

Biotinlylation experiments were performed as decribed in [14] using sulfo-NHC-LC-Biotin in PBS (Pierce). Biotinylated ALK was detected with IRDye800CW-coupled streptavidin (1/10000, Rockland, ME, USA), and total ALK with anti-ALK REAB and secondary anti-rabbit IgG IRDye700DX-coupled antibodies.

### Immunofluorescence

All immunofluoresence studies were performed at the Institut du Fer à Moulin imaging facilities. CHO cells were transfected with pcDNA3-ALK WT or F1174L and then cultured for two days. Media were replaced with cold media containing 1 µg/ml of mAb (46 or 30) and/or transferrin coupled to Alexa Fluor 546 at 50 µg/ml and placed at 4°C for 10 min. Cells were pre-treated 30 min 100 µM of Monensin prior incubation with mAb at 4°C for 10 minutes. Cold media with ligand were removed and replaced by hot media and cells were replaced at 37°C in a time course manner (15–180 min). Cells were fixed with paraformaldehyde for 15 min at room temperature and then permeabilized in PBS 0.5%.Triton X100 5 min. Fixation was quenched with PBS-Glycine 0.1 M for 15 min, and then cells were blocked with PBS-BSA 1.5% during 1 h. ALK bound mAbs were then detected with anti-mouse IgG coupled to Alexa Fluor 488 1/400, molecular probes) during 1 hour. After washing in PBS, cells were mounted in Vectashield (Vector lab). Confocal laser microscopy was performed using a TCS SP2 confocal microscope (Leica). Images were assembled using Adobe Photoshop software.

### RT-PCR and sequencing

Total RNA were extracted from SH-SY5Y or IMR-32 cells with RNeasy extraction kit (Quiagen) according to manufactor instruction. cDNA was synthetized from RNA by reverse transcriptase and oligodT primer. ALK cDNA sequence comprising c3353 was then amplified by PCR using Taq DNA polymerase and the following primers

Primer F: 5′ATGACTGTGTTACCGCCGGAAGC-3′

Primer R: 5′TTAACTGGCAAGCATGGCACAGC-3′.

Presence of ALK mutation was determined by PCR products sequencing.

### Mass spectrometry analysis

a) 1D SDS-PAGE separation and Nano-LC-MS/MS Analysis

After immunoprecipitation samples were separated on a 4–12% acrylamide SDS-PAGE gel. Proteins were visualized by coomassie blue staining (Labsafe GEL Blue, G-Biosciences). Gel slices were reduced, alkylated and subjected to digestion with trypsin (Sequence Grade Modified, Roche Diagnostics) as previously described [31].

The peptides mixtures were analyzed by nano-LC-MS/MS using an Ultimate3000 system (Dionex S. A.) coupled to an LTQ-Orbitrap mass spectrometer (Thermo Fisher Scientific. Bremen, Germany). Data-dependent acquisition was performed on the LTQ-Orbitrap mass spectrometer in the positive ion mode. Survey MS scans were acquired in the orbitrap on the 400–1100 m/z range with the resolution set to a value of 60 000. Each scan was recalibrated in real time by co-injecting an internal standard from ambient air into the C-trap (‘lock mass option’). The 5 most intense ions per survey scan were selected for CID fragmentation and the resulting fragments were analyzed in the linear trap (LTQ). The parent mass list included the m/z value of the two peptides at 497.28 Da and 514.28 Da. Target ions already selected for MS/MS were dynamically excluded for 60 s. Data were acquired using the Xcalibur software (version 2.0.5).

b) Database Search and Data Analysis

The resulting spectra were then analyzed via the Mascot™ Software created with Proteome Discoverer (version: 1.2.0.92, Thermo Scientific) using the “homo sapiens” (human) non-redundant database of the National Center for Biotechnology Information (National Library of Medicine, Bethesda 28^th^ February 2011, 235506 sequences). Carbamidomethylation of cysteines, oxidation of methionine and protein N-terminal acetylation were set as variable modifications for all Mascot searches. Specificity of trypsin digestion was set for cleavage after Lys or Arg except before Pro, and one missed trypsin cleavage site was allowed. The mass tolerances in MS and MS/MS were set to 2 ppm and 0.8 Da respectively, and the instrument setting was specified as “ESI-Trap.” All data were validated by using myProMS (Proteomics, 2007).

c) Label-free Quantification

Quantification of peptide FNHQNIVR (wt) and LNHQNIVR (mutant) in band 220 kD and 140 kD. To quantify the ALK proteins (WT/F1174L), the extracted ion chromatogram (XIC) signal of the well characterized tryptic peptide ions (wt and mutant) was manually extracted from the MS survey of nano-LC-MS/MS raw files using the Xcalibur software. XIC areas were integrated in Xcalibur under the QualBrowser interface using the ICIS algorithm. Mean values and S.D. were calculated for quadruplicate measurements.

## Supporting Information

Figure S1 (**A**) Shown are the MS/MS fragmentation spectra of the tryptic peptides FNHQNIVR (wt, aa 1174–1181) and LNHQNIVR (mutant) of Alk. (**B**) Signal abundance plot of the synthetic wt and mutant peptides at different concentrations - synthetic mutant (LNHQNIVR) black circle and synthetic wt (FNHQNIVR) red triangle were mixed in different ratios (from 0.5 fmol to 50 fmol on column) and analyzed on a QSTAR in MS mode. The coefficient of variation in ionization efficiency of the wt/mutant was used to correct the relative abundance correspond to 0.845.
(TIF)

Figure S2 **Cell surface proteins of SH-SY5Y cells were biotinalyted as described in Methods.** ALK immunoprecipitates from 1.5 mg of total cell lysate proteins were immunoblotted with Strepatividine coupled to Dylight 800 for cell surface ALK detection (in green) and total ALK were detected with polyclonal anti-ALK (REAB) and secondary anti-rabbit IgG coupled to IRdye700 (in red).
(TIF)
