# Supplementary figures and images for: Internalization and Down-Regulation of the ALK Receptor in Neuroblastoma Cell Lines upon Monoclonal Antibodies Treatment
Source: PLoS One. 2012 Mar 30;7(3):e33581. doi: 10.1371/journal.pone.0033581 (PMC3316580; doi:10.1371/journal.pone.0033581)

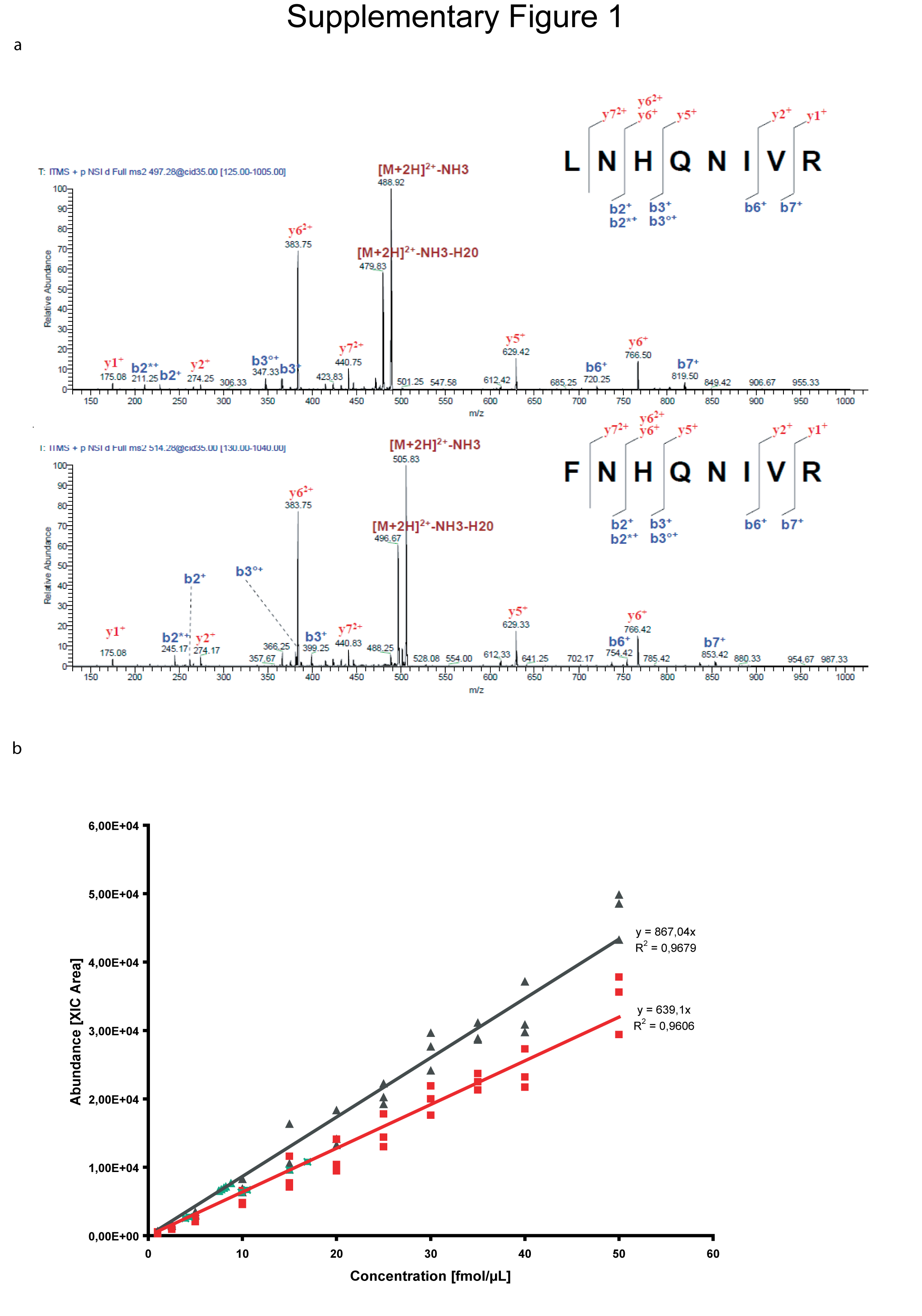

Supplement: Figure S1 — (A) Shown are the MS/MS fragmentation spectra of the tryptic peptides FNHQNIVR (wt, aa 1174–1181) and LNHQNIVR (mutant) of Alk. (B) Signal abundance plot of the synthetic wt and mutant peptides at different concentrations - synthetic mutant (LNHQNIVR) black circle and synthetic wt (FNHQNIVR) red triangle were mixed in different ratios (from 0.5 fmol to 50 fmol on column) and analyzed on a QSTAR in MS mode. The coefficient of variation in ionization efficiency of the wt/mutant was used to correct the relative abundance correspond to 0.845. (TIF) [file pone.0033581.s001.tif]

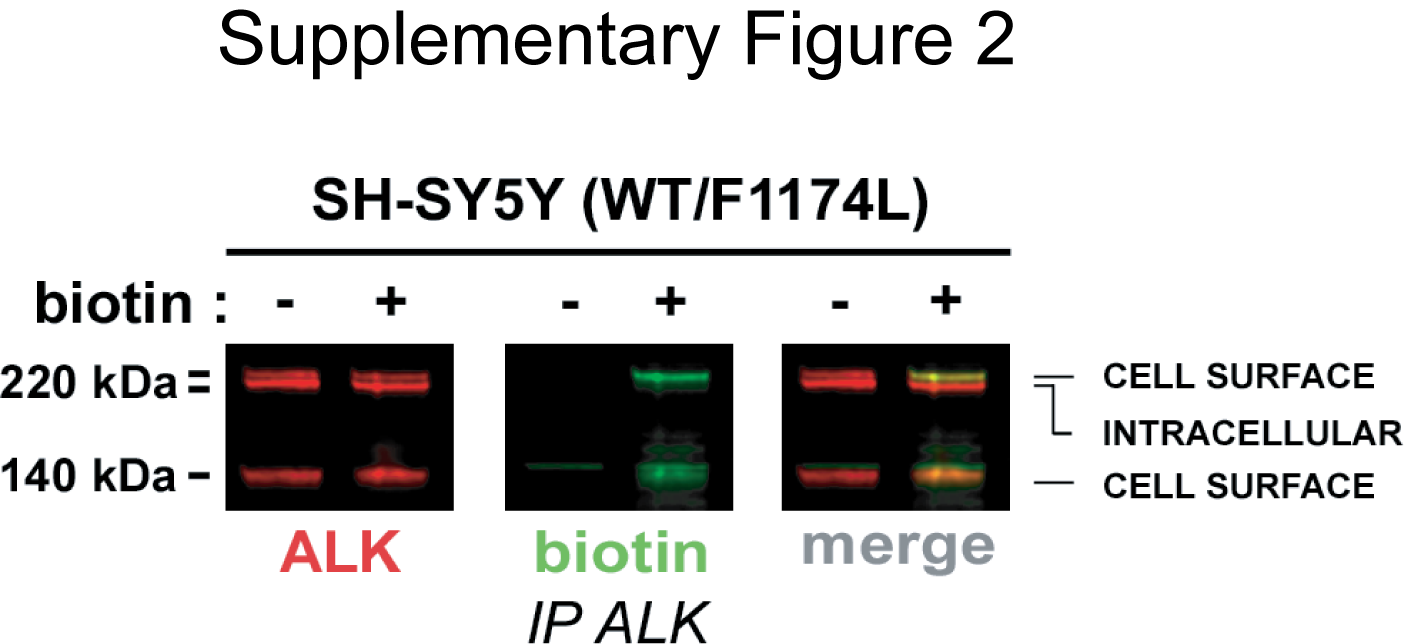

Supplement: Figure S2 — Cell surface proteins of SH-SY5Y cells were biotinalyted as described in Methods. ALK immunoprecipitates from 1.5 mg of total cell lysate proteins were immunoblotted with Strepatividine coupled to Dylight 800 for cell surface ALK detection (in green) and total ALK were detected with polyclonal anti-ALK (REAB) and secondary anti-rabbit IgG coupled to IRdye700 (in red). (TIF) [file pone.0033581.s002.tif]
